# Insane Soldiers and the Law

**Published:** 1917-03-24

**Authors:** 


### The Hospital

The Workers' Newspaper of Administrative Medicine and Institutional Life, Administration, National Insurance and Health.

No. 1607, Vol. LXI. SATURDAY, MARCH 24, 1917. PRICE ONE PENNY

### INSANE SOLDIERS AND THE LAW.

Much feeling has been aroused in many parts of the country, and especially in Lancashire, over the treatment of insane soldiers. We are not sure that this is wholly justified, though it is born of a very excellent state of mind, which has never existed widely, certainly never nearly so widely, until the present war. A hundred years ago, the private soldier was looked upon in this country very much as he is now regarded in Germany, as cannon-fodder. The Army was recruited, in time of peace, from the very lowest outcasts, from the sweepings of the slums, the workhouses, and the gaols, and was succinctly described by its commander as "the dregs of the earth, officered by the scum." Yet how it fought! The history of fighting contains no record of a battle more bitterly contested or more heroically won than that of Albuera, like Inkerman, a private soldiers' battle. The unfortunates who were wounded in the Peninsula and at Quatre Bras and Waterloo had to endure what was more appalling even than their wounds, that is to say the primitive surgery of the time, in which antiseptics were as unknown as the need for them, and anæsthetics had not yet been discovered. The probing and searching for missiles in the tissues of the body, the opening up of the tissues to get at them and extract them, the trimming off of unsaveable parts, the amputations, and the stitchings and dressings that followed them were all done without the oblivion of the merciful anæsthetic that now deprives operations of nineteen-twentieths of their horrors. Each man waited his turn in sight of the tortures and torments being endured by his predecessor. If the actual experience of fighting is more terrible in these present days, let us be thankful, at least, that the after-experience of the wounded is shorn of the greatest part of its horrors. Add to them the fearful ravages of pyæmia, septicæmia, gangrene, and tetanus, and the general contempt and indifference of the combatant commands for the medical element in the Army, the indifference to its needs, the contempt of its recommendations, and we shall wonder, not at the enormous proportion of the wounded who died of even slight wounds, but at the considerable numbers of the survivors. And what of these survivors? What became of them? When the campaign was over, the Army was disbanded, and the men were left to their own devices, to join the civil population and get their living as best they might. Is it to be wondered at that crime assumed portentous dimensions, that many disbanded soldiers "took to the road," in other words, turned highway robbers, that others followed other criminal pursuits, that the gaols were crammed, that ships stuffed with convicts started for Botany Bay every month, and that ten, fifteen, and twenty felons were hanged at Tyburn every Monday morning? These were mostly the able-bodied, but what of the wounded and maimed? They were left to beg, to tramp the country, and live by mendicancy supplemented by pilfering, and such petty crimes as their mutilated limbs permitted them to commit. Such a state of things seems to us incredible, but that it should seem so is only one more instance of that wonderful increase in humanity, in sympathy, in tenderness for human failings, and above all for human suffering, that began in the first half of the last century, and has gone on increasing ever since, until before this great war it had reached in many people an excess, and had become a mawkish and sickly and sentimental humanitarianism. The war has, to a considerable extent, rectified this, as it has rectified some of the errors of the body politic. There are too many cases of real and intense suffering now for much sympathy to be poured out upon unfit objects, and values have been rectified and adjusted. Hence, when an injustice is committed that would have been thought no injustice a hundred years ago, nay, that would have been considered a wonderful and superfluous benevolence, the whole country is up in arms about it.

The matter is this: disabled soldiers are discharged from the Army with a pension that is enough to keep them and their families from want. But if this disablement is a mental disablement, they are sent into asylums to be cared for and treated. There is no hardship in this. The asylums are pauper asylums it is true, but they are the same asylums to which men of the same class have to go if they become insane in civil life. So far, then, there is no ground of complaint. But when they are passed into these asylums the men become subject to the lunacy law, under which they are charged, if they have the means, with the cost of their maintenance. This runs away with half or more than half of their little pensions, and leaves their wives and families with a very insufficient pittance to live upon. It is such a manifest and palpable injustice, and has aroused so much clamour, that it will very soon be rectified now that attention is drawn to it, and Mr. Barnes, the Minister for Pensions, has given a promise to consider the matter sympathetically.

